# 377. SARS-CoV-2 Genomic Surveillance Reveals Little Spread Between a Large University Campus and the Surrounding Community

**DOI:** 10.1093/ofid/ofab466.578

**Published:** 2021-12-04

**Authors:** Andrew Valesano, William Fitzsimmons, Christopher Blair, Robert Woods, Julie Gilbert, Dawn Rudnik, Lindsey Mortenson, Joshua G Petrie, Emily T Martin, Adam Lauring

**Affiliations:** University of Michigan, Ann Arbor, Michigan

## Abstract

**Background:**

Understanding SARS-CoV-2 transmission dynamics is critical for controlling and preventing outbreaks. The genomic epidemiology of SARS-CoV-2 on college campuses has not been comprehensively studied, and the extent to which campus-associated outbreaks lead to transmission in nearby communities is unclear. We used high-density genomic surveillance to track SARS-CoV-2 transmission across the University of Michigan-Ann Arbor campus and Washtenaw County during the Fall 2020 semester.

**Methods:**

We retrieved all available residual diagnostic specimens from the Michigan Medicine Clinical Microbiology Laboratory and University Health Service that were positive for SARS-CoV-2 from August 16^th^ – November 25^th^, 2020 (n = 2245). We extracted viral RNA, amplified the SARS-CoV-2 genome by multiplex RT-PCR, and sequenced these amplicons on an Illumina MiSeq. We applied maximum likelihood phylogenetic analysis to whole genome sequences to define and characterize transmission lineages.

**Results:**

We assembled complete viral genomes from 1659 individual infections, representing roughly 25% of confirmed cases in Washtenaw County across the fall semester. Of these cases, 468 were University of Michigan students. Phylogenetic analysis revealed 203 genetically distinct introductions of SARS-CoV-2 into the student population, most of which were singletons (n = 171) or small clusters of 2 – 8 students. We identified two large SARS-CoV-2 transmission lineages (115 and 73 students, respectively), including individuals from multiple on-campus residences. Viral descendants of these student outbreaks were rare, constituting less than 4% of cases in the community.

**Conclusion:**

We identified many SARS-CoV-2 transmission introductions into the University of Michigan campus in Fall 2020. While there was widespread transmission among students, there is little evidence that these outbreaks significantly contributed to the rise in COVID-19 cases that Washtenaw County experienced in November 2020.

**Disclosures:**

**Adam Lauring, MD, PhD**, **Roche** (Advisor or Review Panel member) **Sanofi** (Consultant)

